# MHC Universal Cells Survive in an Allogeneic Environment after Incompatible Transplantation

**DOI:** 10.1155/2013/796046

**Published:** 2013-10-09

**Authors:** Constança Figueiredo, Dirk Wedekind, Thomas Müller, Stefanie Vahlsing, Peter A. Horn, Axel Seltsam, Rainer Blasczyk

**Affiliations:** ^1^Institute for Transfusion Medicine, Hannover Medical School, Lower Saxony, 30625 Hannover, Germany; ^2^Institute for Laboratory Animal Science, Hannover Medical School, Lower Saxony, 30625 Hannover, Germany; ^3^Institute for Transfusion Medicine, University Hospital Essen, 45147 Essen, Nordrhein-Westfalen, Germany; ^4^German Red Cross, Blood Services NSTOB, Institute Springe, Lower Saxony, 31832 Springe, Germany

## Abstract

Cell, tissue, and organ transplants are commonly performed for the treatment of different diseases. However, major histocompatibility complex (MHC) diversity often prevents complete donor-recipient matching, resulting in graft rejection. This study evaluates in a preclinical model the capacity of MHC class I-silenced cells to engraft and grow upon allogeneic transplantation. Short hairpin RNA targeting **β**2-microglobulin (RN_sh**β**2m) was delivered into fibroblasts derived from LEW/Ztm (RT1^l^) (RT1-A^l^) rats using a lentiviral-based vector. MHC class I (RT1-A-) expressing and -silenced cells were injected subcutaneously in LEW rats (RT1^l^) and MHC-congenic LEW.1W rats (RT1^u^), respectively. Cell engraftment and the status of the immune response were monitored for eight weeks after transplantation. In contrast to RT1-A-expressing cells, RT1-A-silenced fibroblasts became engrafted and were still detectable eight weeks after allogeneic transplantation. Plasma levels of proinflammatory cytokines IL-1**α**, IL-1**β**, IL-6, TNF-**α**, and IFN-**γ** were significantly higher in animals transplanted with RT1-A-expressing cells than in those receiving RT1-A-silenced cells. Furthermore, alloantigen-specific T-cell proliferation rates derived from rats receiving RT1-A-expressing cells were higher than those in rats transplanted with RT1-A-silenced cells. These data suggest that silencing MHC class I expression might overcome the histocompatibility barrier, potentially opening up new avenues in the field of cell transplantation and regenerative medicine.

## 1. Introduction

Regenerative medicine is a new field of research emerging in response to the shortage of organs, tissues, and cells for transplantation and treatment of degenerative diseases [1]. However, the development of therapeutic approaches in this field is often based on the use of allogeneic products. Immunogenicity is a major obstacle to the successful use of these products for allogeneic transplantation. Even autologous cells—either genetically modified adult cells or induced pluripotent stem (iPS) cells—are targeted by the immune system after transplantation. The major histocompatibility complex (MHC) is the most relevant genomic region responsible for transplant rejection. Human MHC proteins are referred to as human leukocyte antigens (HLA) because they were first discovered on leukocytes. HLA compatibility plays a pivotal role in the success of allogeneic transplantation, and the number of donor-recipient HLA mismatches is associated with the severity of graft rejection and the transplant survival rate [2–5].

A continuously growing number of new HLA alleles have been identified by molecular genetic analysis in the last two decades, reflecting the great diversity of the HLA loci. Because an extremely large pool of donors is needed to find an unrelated HLA-matched donor for a given individual, it is usually impossible to find a complete HLA-matched donor, especially for patients with rare HLA alleles. Improvements in immunosuppressive therapy have reduced the degree of T-cell-mediated immune response to grafts, resulting in an increase in overall graft survival and a decrease in acute rejection [6–8]. Nonetheless, rejection due to antibody-mediated graft injury resulting from B-cell responses to mismatched human HLA antigens remains a severe problem. Anti-HLA class I antibodies are involved in acute rejection, whereas anti-HLA class II antibodies are of major importance in late rejection. HLA-A, HLA-B, and HLA-DR compatibility is therefore essential to reduce the number of mismatched T- and B-cell determinants. Furthermore, long-term immunosuppression increases the patient's susceptibility to cancer and opportunistic infections [9–11].

RNA interference (RNAi) is now commonly used to investigate cellular or molecular mechanisms, and the pharmaceutical industry has recognized RNAi as a powerful therapeutic tool for the treatment of both viral infections and diseases caused by the abnormal expression of certain genes [12]. RNAi is a process whereby double-stranded RNA induces sequence-specific degradation of homologous mRNA [13]. The main diseases treated using RNAi gene therapy include hepatitis B, human immunodeficiency virus (HIV) infection [14], cancer [15, 16], neurodegenerative disorders [17], ocular diseases [18], respiratory diseases [19], and arthritis [20]. We have previously described the feasibility of silence HLA class I and class II expression using RNAi technology [21–23]. In addition, other groups have knockedout the expression of HLA class I using Zink-finger nucleases. Furthermore, we and others have shown the feasibility to generate HLA universal (HLA-silenced) cells derived from CD34+ progenitor cells, iPS and ESCs [24]. However, the capacity of HLA-silenced cells to escape the allogeneic immune response was only tested *in vitro*. In this study, we evaluate the capacity of HLA universal cells to survive in a fully allogeneic environment in a rat transplantation model. Rat models are the most widely used experimental transplantation models. As in humans, MHC known as the RT1 complex regulates immune responses to natural proteins. Human MHC class I molecules are highly homologous to rat MHC class I molecules, which are designated RT1-A and RT1-C/-E/-M. Several haplotypes have been detected within the RT1-A region [25]. Therefore we have selected this animal model to investigate whether HLA-silenced adult cells have a superior capacity to prevent and escape the allogeneic immune response. 

## 2. Material and Methods

### 2.1. Experimental Animals

Inbred (LEW) and coisogenic inbred (LEW.1W) rats differ only in their MHC haplotype (RT1^l^: RT1-A^l^, RT1-B/D^l^, RT1-C^l^ versus RT1^u^: RT1-A^u^, RT1-B/D^u^, and RT1-C^u^). All strains used at our facility are regularly subjected to genetic testing in order to exclude the possibility of genetic contamination and to verify the authenticity of the strains. The LEW and LEW.1W rats were maintained in a minimally sustained facility that is regularly monitored for murine pathogens according to FELASA recommendations. Both strains were kept under identical conditions with 10 : 14 h light-dark cycles in type IV Macrolon cages with free access to water and sterilized standard laboratory chow (Diet number 1324, Altromin, Detmold, Germany).

Appropriate ethical and regulatory approval for use of the animals and animal-derived tissues was obtained.

### 2.2. Short Hairpin RNA Design and Vector Production

Regions sensitive for short hairpin RNA (shRNA) were identified on *β*2-microglobulin (*β*2m) transcripts from brown rats (*Rattus norvegicus*). shRNA sequences silencing *β*2m expression were designed using a web-based algorithm (siRNA Target finder; http://www.ambion.com/). pLVTHM, psPAX2, and pMD2G vectors were kindly provided by Didier Trono of Geneva, Switzerland. Oligonucleotides containing the selected shRNA sequences were cloned into pLVTHM. RT1 cells showing a reduction by up to 70% in RT1-A protein expression in comparison to the cells transduced with a vector encoding for a control shRNA (shNS) were produced by the delivery of shRNA_1. A reduction higher than 70% was achieved by the delivery of shRNA_2. This shRNA_2 sequence was selected to produce the cell line for the transplantation experiments described in this study and received the name RN_sh*β*2m (See Supplementary Material available online at http://dx.doi.org/10.1155/2013/796046). 

Lentiviral particles were produced by transfecting 5 × 10^6^ human embryonic kidney (HEK)293T cells using Lipofectamine 2000 (Invitrogen, Carlsbad, CA) according to the manufacturer's instructions. Transfection was performed using 9 *μ*g of psPAX2 (plasmid containing the *gag *and *pol *genes and encoding the structural proteins and the reverse transcriptase), 3 *μ*g of pMD2G (plasmid encoding vesicular stomatitis virus G envelope protein), and 10 *μ*g of pLVTHM (plasmid encoding the shRNA sequences). After 16 hours of transfection, the cells were washed and incubated with complete Dulbecco's Modified Eagle's Medium (DMEM, LONZA, Verviers, Belgium) supplemented with 10% (vol/vol) fetal calf serum (FCS) and 1% (vol/vol) glutamine. Virus supernatants were collected 48 hours after transfection and passed through a 0.45 mm filter (Millipore, Billerica, MA). Virus supernatants were concentrated overnight by centrifugation at 12000 g and 4°C.

### 2.3. Cell Culture

A LEW fibrosarcoma cell line (RT1, ECACC 94011416) was stably transfected with pcDNA3.1-V5/His (Invitrogen, Groningen, The Netherlands), a plasmid-based vector encoding for the firefly luciferase gene sequence, and cultured in Eagle's Minimum Essential Medium (LONZA, Verviers, Belgium) supplemented with 2 mM glutamine (C.C. Pro, Neustadt, Germany), 1% (vol/vol) nonessential amino acids (Invitrogen/GIBCO, Auckland, New Zealand), and 5% (vol/vol) FCS (LONZA). HEK cells were cultured in DMEM (LONZA) supplemented with 2 mM glutamine (C.C. Pro, Neustadt, Germany), 1% (vol/vol) nonessential amino acids (Invitrogen/GIBCO, Auckland, New Zealand), and 10% (vol/vol) FCS (LONZA).

### 2.4. Cell Transduction

RT1 fibrosarcoma cells (1 × 10^6^) were resuspended in 1 mL of concentrated viral supernatant and plated on six-well dishes. Cell transduction was performed in the presence of 8 mg/mL protamine sulfate (Sigma-Aldrich) at 37°C for 8 hours, after which the cells were supplied with fresh medium. On day 4, RT1-A expression was evaluated by flow cytometry after staining with a phycoerythrin- (PE-) labeled mouse anti-rat RT1-A antibody (BD Bioscience, Erembodegem, Belgium). PE-conjugated mouse immunoglobulin (Ig)G antibody (BD Bioscience) was utilized for isotype control. *β*2m transcript levels were determined by real-time polymerase chain reaction (PCR) on days 4, 8, and 30.

### 2.5. Real-Time PCR

Total RNA from transduced and nontransduced fibroblasts was isolated 4 days after transduction or immediately before cell transplantation and used in reverse transcription reactions to cDNA (High Capacity cDNA Reverse Transcription Kit, Applied Biosystems, Foster City, CA). Fifty nanograms of total RNA were used in each reverse transcription reaction. *β*2-microglobulin and *β*-actin RNA levels were determined by two-step real-time PCR (TaqMan Gene Expression Master Mix, Applied Biosystems) using amplification primers and FAM-labeled TaqMan minor groove-binding probes for *β*2-microglobulin (5′-GCCATCCACCGGAGAATG-3′ and 5′-GGTGGAACTGAGACACGTAGCA-3′; 5′-FAM-AAGCCCAACTTCC-3′) and *β*-actin (5′-TTCAACACCCCAGCCATGT-3′ and 5′-CAGAGGCATACAGGGACAACAC-3′; 5′-FAM-CGTAGCCATCCAGGC-3′). The constitutively expressed *β*-actin gene was used as the control for normalization of cDNA levels. Thermal cycling was performed on a commercially available real-time PCR system (StepOne Plus, Applied Biosystems) at 95°C for 10 minutes followed by 40 cycles at 95°C for 15 seconds and 60°C for 1 minute.

### 2.6. Cell Transplantation

LEW rat-derived fibrosarcoma cells were transplanted into LEW rats (syngeneic transplantation) or LEW.1W rats (allogeneic transplantation), respectively. One million fibrosarcoma cells were injected subcutaneously in the left hind leg of each animal. Groups of eight animals each received either RT1-A-expressing or RT1-A-silenced fibrosarcoma cells. 

### 2.7. Evaluation of Tumor Growth by Palpation and Collection of Tumor Cells after Transplantation

After cell transplantation, tumor formation and growth were monitored weekly by palpation and measured weekly using a vernier caliper. Tumor volume (*V*) was calculated using the formula *V* = *π*.ri.r0.h. In addition, a tumor biopsy was performed at week 2 after transplantation, and samples of tumor cells were collected at the time sacrificing the animals (week 8).

### 2.8. *In Vivo* Bioluminescence Imaging

The rats were anesthetized with ketamine (100 mg/kg intraperitoneally) and xylazine (10 mg/kg 226 intraperitoneally), and an aqueous solution of D-luciferin (150 mg/kg) was injected subcutaneously 5 minutes before bioluminescence imaging. The animals were then placed in a dark chamber of the charge-coupled device camera (IVIS200, Xenogen, Cranbury, NJ, USA), and grayscale body surface reference images (digital photographs) were taken under weak illumination. The light source was switched off, and photons emitted from luciferase-expressing cells within the body and transmitted through the rat tissues were quantified over defined times of up to 5 minutes using Living Image software (Xenogen Biosciences, Cranbury, NJ) as an overlay on Igor Pro (WaveMetrics, Seattle, WA, USA). For anatomical localization, a pseudocolor image representing light intensity (blue, least intense; red, most intense) was generated in Living Image and superimposed over the grayscale reference image. Quantified luminescence consists of averaged photon radiance on the body surface and is expressed as photons/sec/cm^2^/sr where sr: steradian.

### 2.9. Immunohistochemistry Analysis

Tissue generated by growing RT1-A-expressing and RT1A-silenced cells was obtained during pathological analysis and immediately fixed in 4% paraformaldehyde for 48 h, washed twice in phosphate-buffered saline (PBS), embedded in paraffin, and mounted on SuperFrost slides. Embedded tissue sections were incubated in histolene for 10 min to remove the paraffin, rehydrated in decreasing concentrations of alcohol (100, 90, 80, 70, and 50% ethanol for 10 min each), and finally treated with dH_2_O for 10 min. Staining was performed according to the DAKOLSAB System HRB procedure. Primary antibodies were used according to the manufacturer's recommendations (1 : 100), and peroxidase block was applied for 10 min. Slides were blocked for 1 h at room temperature with 5% donkey serum, phosphate-buffered saline, 0.1% bovine serum albumen (BSA), and 0.1% Triton-X-100 and drained and incubated overnight with primary rat natural killer (NK) cell-specific antibody (kindly provided by Dr. Joachim Hundrieser, Center of Surgery, Hannover Medical School) at 4°C in the presence of 1% BSA. Slides were rinsed 2 × 5 min with Tris-buffered saline (TBS) + 0.1% Triton and horseradish peroxidase-conjugated antibodies were added 1 : 200 to TBS + 1% BSA for 1 h at room temperature. After three washing steps in PBS, the sections were embedded in Mowiol/DABCO and analyzed. Pictures were taken with a Biozero BZ-8000 fluorescence microscope (Keyence GmbH, Neu-Isenburg, Germany).

### 2.10. T-Cell Isolation

Peripheral blood samples were collected from the rat donors after transplantation of RT1-A-expressing and -silenced fibrosarcoma cells. Peripheral blood mononuclear cells obtained from these donors were separated by Lymphosep (C.C. Pro) gradient centrifugation followed by T-cell isolation (anti-T-cell OX52 isolation kit, Miltenyi Biotec, Bergisch Gladbach, Germany). T-cell purity was confirmed by flow cytometry using a PE-conjugated anti-CD3 antibody (BD Bioscience).

### 2.11. *In Vitro* T-Cell Proliferation Assays

T cells derived from LEW and LEW.1W rats transplanted with RT1-A-expressing or -silenced fibrosarcoma cells were labeled with carboxyfluorescein succinimidyl ester (CFSE) as previously described [26]. One hundred thousand CFSE-labeled T cells were cocultured with 20 × 10^4^ irradiated RT1-A-expressing fibrosarcoma cells in a 96-well plate for 8 days. T-cell proliferation was analyzed by flow cytometry.

### 2.12. *In Vitro* Evaluation of NK Cell Activity

RT1 fibroblasts expressing different levels of RT1-A were produced by lentiviral-mediated delivery of shRNAs with different efficiencies in knocking down *β*2-microglobulin. Two shRNA sequences (shRNA_1 and shRNA_2) were used (Table S1). Several RT1 fibrosarcoma cell lines were produced showing reductions on RT1-A expression between 40% and more than 90% as measured by flow cytometric analysis. NK cells of 4 rats were isolated from peripheral blood mononuclear cells (PBMCs) by fluorescence-activated cell sorting after cell staining with a phycoerythrin-(PE-) conjugated mouse anti-rat CD161 (BD Biosciences). Cytotoxic assays were performed by exposing NK cells to fibroblast cells showing different levels of RT1-A expression at an effector: target ratio 5 : 1 for 6 h. Target cell lysis was detected using 7-amino-actinomycin D (7-AAD). To evaluate the differences between the means of the groups, two-way ANOVA and Bonferroni post tests were performed. Both analyses were carried out using GraphPad Prism v.5.02 (San Diego, CA). Statistically significant differences are shown with asterisks (*P < 0.05, **P < 0.01, ***P < 0.001).

## 3. Results

### 3.1. Silencing of RT1-A Expression

**Figure 1:**
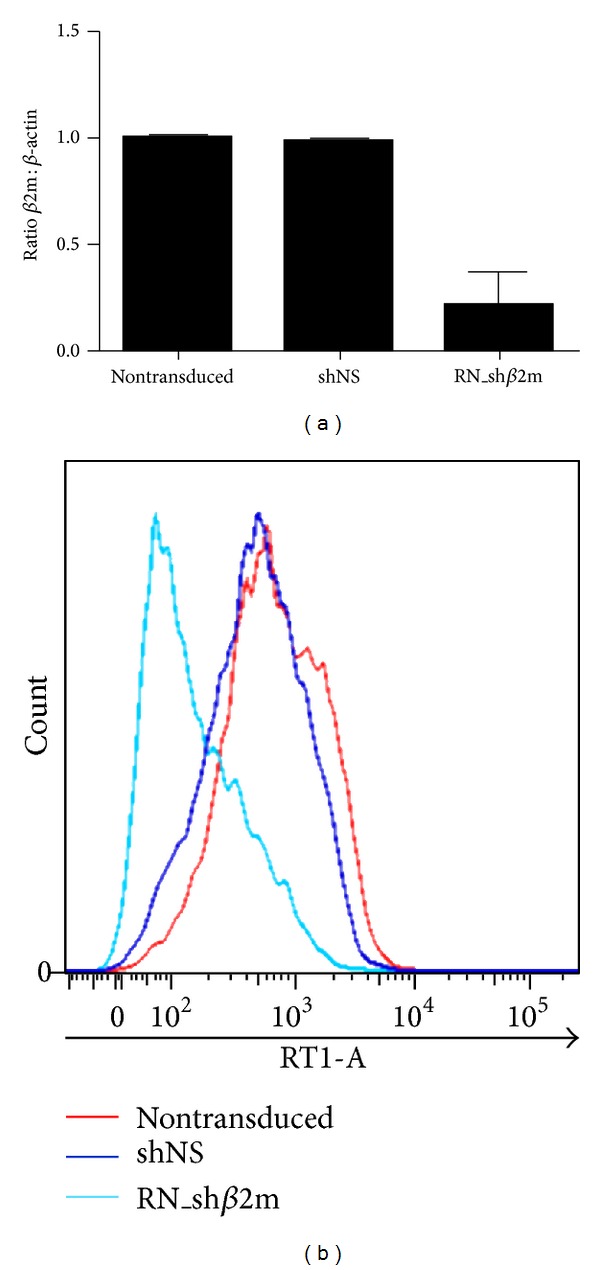
Silencing of RT1-A expression in the LEWis (LEW) rat-derived fibrosarcoma cell line. The fibrosarcoma cell line was transduced using either lentiviral vectors encoding for nonspecific short hairpin RNA (shNS) or a *β*2-microglobulin (*β*2m-) specific short hairpin RNA (RN_sh*β*2m). (a) Relative transcript levels of *β*2m in fibrosarcoma cells, as determined by real-time PCR 4 days after transduction. (b) Flow cytometry analysis of RT1-A expression on nontransduced fibrosarcoma cells and after the delivery of shNS or RN_sh*β*2m.

Rat LEW-derived RT1 fibrosarcoma cells expressing firefly luciferase were transduced with either a lentiviral vector encoding for nonspecific shRNA or *β*2m-specific shRNA (Table S1). This cell line is a susceptible target to T and NK cell activity, and it is of great advantage in transplant rejection models. A transduction efficiency of 95 ± 4% was achieved. Transduction of RT1 fibroblasts with the vector coding for *β*2m-specific shRNA (RN_sh*β*2m) caused an 82% reduction of *β*2m mRNA in comparison to nonmodified cells. This resulted in an up to 80 ± 7% decrease in RT1-A protein expression on the cell surface. In comparison to nontransduced cells, delivery of nonspecific shRNA (shNS) into RT1 fibroblasts did not significantly affect *β*2m mRNA levels. Fibroblasts expressing shNS showed 98% of the RT1-A expression measured on nonmodified cells (Figure 1).

### 3.2. Evaluation of Cell Engraftment and Proliferative Capacity

**Figure 2:**
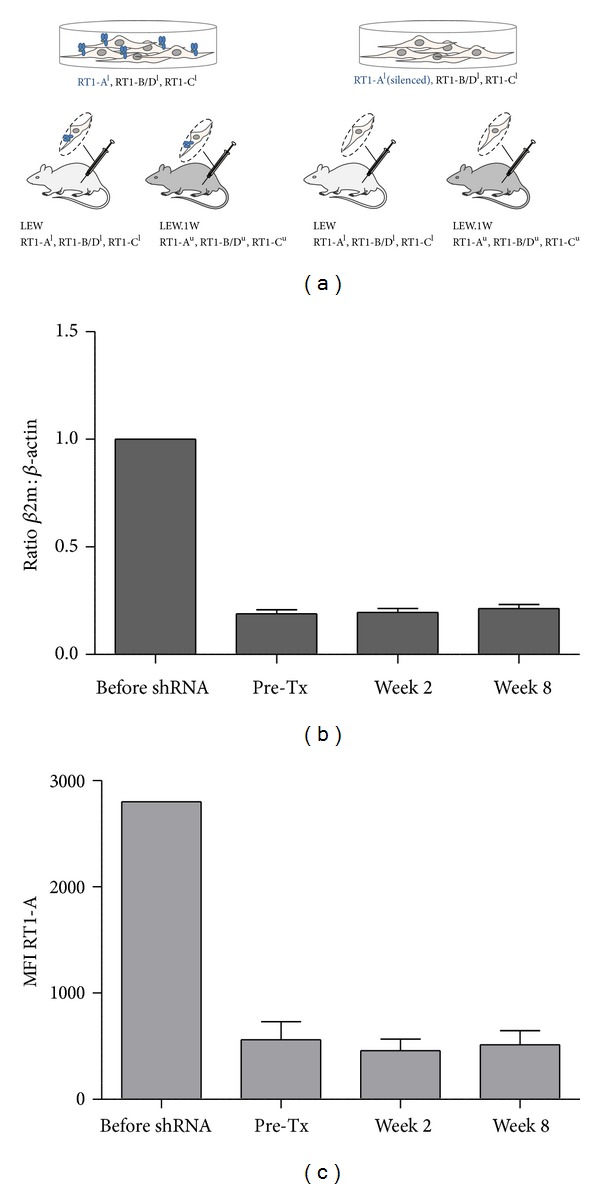
Syngeneic and allogeneic transplantation models of stably MHC class I-silenced cells. (a) Fibrosarcoma cells expressing RT1-A molecules and control shRNA (shNS) or RT1-A-silenced fibrosarcoma cells derived from LEWis (LEW) rats after delivery of *β*2-microglobulin-specific shRNA (RN_sh*β*2m) were injected subcutaneously into the left hind leg of LEW rats (haplotype-matched setting) or LEW.1W rats (haplotype-mismatched setting). Histograms depict *β*2-microglobulin levels measured by real-time PCR (b) and MHC class I expression represented as mean fluorescence intensity (MFI) (c) on the fibroblast cells before the delivery of the shRNAs, immediately before transplantation, and two and eight weeks after transplantation.

**Figure 3:**
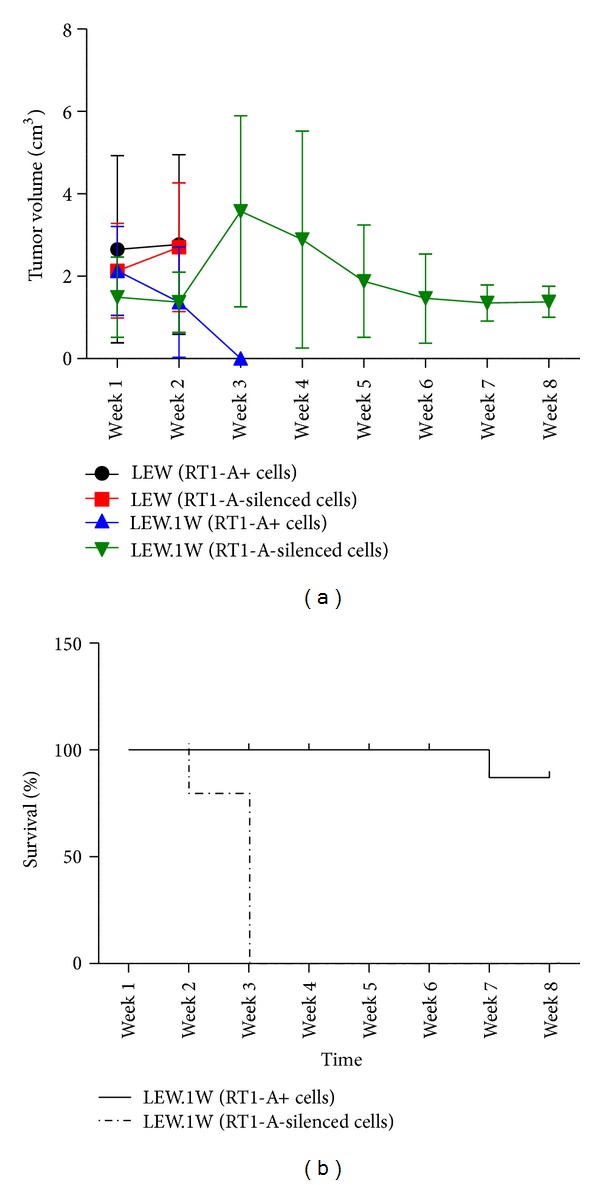
RT1-A silencing promotes graft survival in allogeneic transplantation. (a) Mean tumor volume. (b) Percentage of graft survival after allogeneic transplantation. Cell engraftment and tumor growth were monitored for 8 weeks after allogeneic transplantation by palpation and bioluminescence. All animals in the haplotype-matched transplantation groups receiving RT1-A-expressing or RT1-A-silenced cells were sacrificed 2 weeks after transplantation to prevent morbidity due to excessive tumor growth.

**Figure 4:**
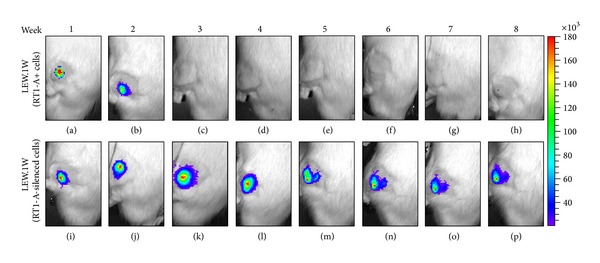
*In vivo *engraftment and viability of RT1-A-expressing or -silenced LEW rat-derived fibroblasts after subcutaneous injection into LEW.1W rats. *In vivo *optical imaging analysis of the injection site in representative LEW.1W rats. The reference color bar indicates high levels of bioluminescence in red and low levels in blue.

The engraftment and proliferative capacities of RT1-A-expressing versus RT1-A-silenced fibrosarcoma cells were evaluated by palpation after syngeneic and allogeneic transplantation of the cells (Figure 2(a)). *β*2-microglobulin transcript levels remained downregulated as detected at weeks 2 and 8 after cell transplantation (ratio *β*2m: *β*-actin week 2: 0.20 ± 0.07; week 8: 0.21 ± 0.06) accordingly to the levels measured immediately before transplantation (0.19 ± 0.05). Similarly, RT1-A expression levels remained silenced after transplantation (MFI week 2: 459.7 ± 107.5, week 8: 513.5 ± 133.0) as compared to RT1-A expression level detected prior to transplantation (552.2 ± 169.1) (Figures 2(b) and 2(c)). Tumor growth was measured weekly for up to eight weeks. Tumor volumes were measured as mean ± standard deviation (cm^3^) in eight animals per group. Two weeks after transplantation into LEW rats (syngeneic model), tumor volumes of 2.77 ± 2.18 cm^3^ and 2.70 ± 1.56 cm^3^ were detected in the animals injected with RT1-A-expressing and -silenced cells, respectively. The animals were sacrificed at that time to prevent pain and morbidity due to excessive tumor growth. Tumor volumes were much smaller after allogeneic transplantation into LEW.1W rats. In these animals, mean tumor volumes of 1.37 ± 1.34 cm^3^ and 1.37 ± 0.73 cm^3^ were measured two weeks after transplantation of RT1-A-expressing or silenced fibrosarcoma cells. No tumor was detectable three weeks after transplantation in LEW.1W rats receiving RT1-A-expressing fibrosarcoma cells. In those receiving RT1-A-silenced cells, the mean tumor volume increased to 3.58 ± 2.32 cm^3^ three weeks after injection and then decreased to 2.89 ± 2.63 cm^3^ four weeks after injection and to 1.88 ± 1.36 cm^3^ five weeks after injection. Similar volumes were still detectable eight weeks after transplantation (Figure 3). *In vivo* imaging analyses of the transplanted cells confirmed these results (Figure 4). All animals were sacrificed at that time point.

### 3.3. Detection of T-Cell and NK Cell Infiltration

**Figure 5:**
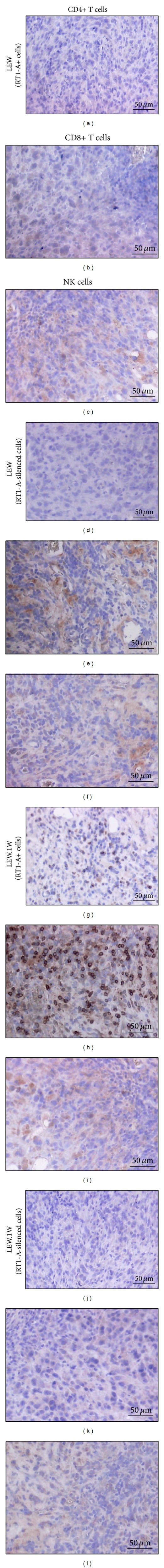
Analysis of tissue infiltration by lymphocytes. T cells and natural killer (NK) cells infiltrated tissues derived from LEW rat cells (RT1-A^l^) after transplantation into LEW and LEW.1W (RT1-A^u^) rats. Scale bar = 50 *μ*m.

Parenchymal leukocyte infiltration after organ transplantation is indicative of organ rejection [27, 28]. Here, immunofluorescence staining was performed to detect the presence of CD4+ and CD8+ T cells. As MHC class I-deficient cells may be targets for NK cells, we also tested for the presence of NK cells in the tissue. CD4+ and CD8+ T-cell infiltration into tissues generated by transplanting RT1-A-expressing cells transplanted into LEW.1W rats was significantly higher than that in tissues generated with RT1-A-silenced cells (Figures 5(a), 5(b), 5(d), 5(e), 5(g), 5(h), 5(j), and 5(k)). No abnormal NK cell infiltration was observed in tissues generated by transplanting RT1-A-silenced cells in an allogeneic setting (Figures 5(c), 5(f), 5(i), and 5(l)).

### 3.4. Cytokine Secretion Profile in Rat Serum after Cell Transplantation

Cytokines are important signaling molecules during rejection episodes [29, 30]. Therefore, we measured cytokine levels in the plasma of rats transplanted with syngeneic or allogeneic fibroblasts with intact or silenced *β*2m expression. Allogeneic transplantation of RT1-A-expressing fibroblasts in LEW.1W rats induced significantly higher IL-1*α* levels (678.3 ± 345.4 pg/mL, *P* < 0.001) than the syngeneic transplantation into LEW rats (7.0 ± 3.2). Nevertheless, allogeneic transplantation of *β*2m-silenced fibroblasts led to a nonsignificant increase in IL-1*α* (36.2 ± 29.8 pg/mL) in LEW.1W rats compared to the levels measured after syngeneic transplantation (IL-1*α*: 7.2 ± 3.7 pg/mL). Similarly, a significant increase in IL-1*β* (80.7 ± 20.3 pg/mL, *P* < 0.01) was detected after allogeneic transplantation of fully RT1-A-expressing fibroblasts. Allogeneic transplantation of *β*2m-silenced fibroblasts into LEW.1W rats caused a slight increase in IL-1*β* secretion (22.7 ± 10.3 pg/mL) relative to levels measured after syngeneic transplantation to LEW rats (15.5 ± 5.0 pg/mL). Allogeneic transplantation of RT1-A-expressing fibroblasts into LEW.1W rats resulted in significantly higher IL-6 levels than syngeneic transplantation of these cells into LEW rats (789.6 ± 111.2 versus 138.9 ± 58.7 pg/mL; *P* < 0.001). However, allogeneic transplantation of *β*2m-silenced fibroblasts and syngeneic transplantation of RT1-A-expressing or -silenced cells resulted in comparable IL-6 expression levels (159.8 ± 123 versus 126.2 ± 73.4 pg/mL). Significantly higher IFN-*γ* secretion (130.9 ± 80.3 pg/mL, *P* < 0.001) was detected after allogeneic transplantation of RT1-A-expressing cells than after syngeneic transplantation (23.2 ± 10.4 pg/mL). Interestingly, injection of RT1-A-silenced cells into allogeneic recipients induced significantly lower secretion of IFN-*γ* (32.1 ± 6.4 pg/mL, *P* < 0.001). Similar results were observed for TNF-*α* secretion. Significantly higher TNF-*α* levels (45.9 ± 18.2 pg/mL, *P* < 0.001) were detected after transplantation of RT1-A-expressing cells into LEW.1W rats versus LEW rats (8.0 ± 1.5 pg/mL). However, serum TNF-*α* levels were significantly lower (10.3 ± 5.2 pg/mL, *P* < 0.001) after allogeneic transplantation of RT1-A-silenced cells (Table 1).

This data suggests that silencing of MHC class I expression in grafted cells does not induce a cytokine storm that might contribute to amplify the immune response against cells from allogeneic sources.

### 3.5. T-Cell Proliferation Assay

**Figure 6:**
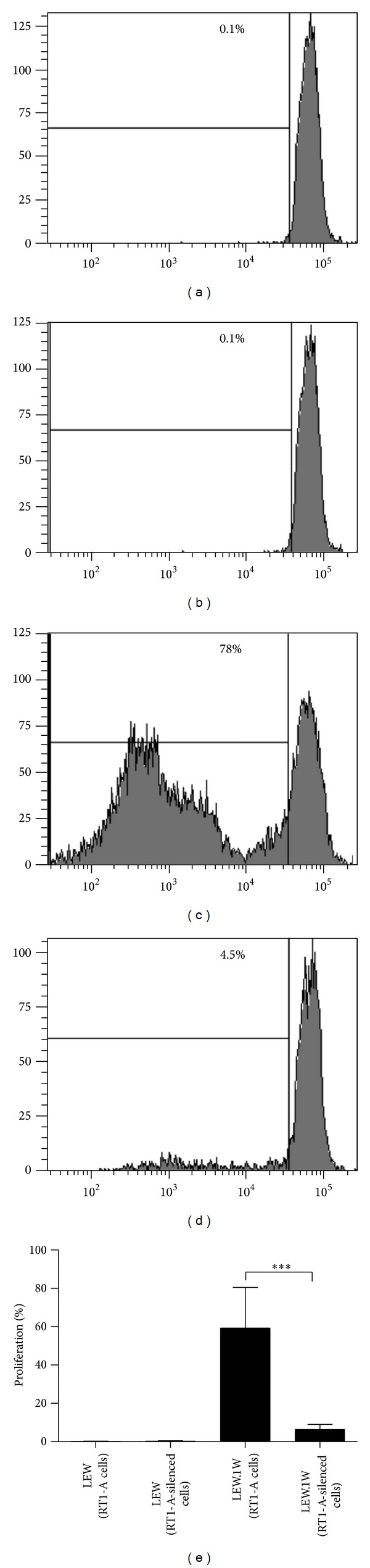
T-cell proliferation assays. Eight weeks after transplantation, T cells were isolated from LEW.1W rats transplanted with either RT1-A-expressing or RT1-A-silenced fibroblasts, stained with CFSE, and cocultured *in vitro *in the presence of RT1-A-expressing fibroblasts for 8 days. ((a)–(d)) Representative experiment for determination of proliferation rates of T cells isolated from (a) LEW rats transplanted with RT1-A-expressing fibroblasts, (b) LEW rats transplanted with RT1-A-silenced fibroblasts, (c) LEW.1W rats transplanted with RT1-A-expressing fibroblasts, and (d) LEW.1W rats transplanted with RT1-A-silenced fibroblasts. Values are represented as mean and standard deviation of five independent experiments. Two-way ANOVA was used to calculate statistically significant differences between groups. Statistical significance is indicated by asterisks (*P < 0.05, **P < 0.01, ***P < 0.001).

Proliferative T-cell alloresponses in mixed lymphocyte reactions indicate a high susceptibility for transplant rejection and are predictive for reduced organ survival. The presence of memory T cells generated *de novo *after exposure to a first allogeneic graft might constitute an important risk for subsequent organ transplantations [31, 32]. Therefore, we performed proliferation assays to compare the capacities of RT1-A-expressing versus RT1-A-silenced cells to induce the expansion of tissue-specific T-cell clones isolated from LEW or LEW.1W rats transplanted with RT1-A-expressing or -silenced cells. 59.3 ± 21.2% of T cells derived from LEW.1W rats transplanted with RT1-A-expressing cells proliferated after *in vitro *exposure to these cells, whereas only 6.4 ± 2.7% of T-cells derived from LEW.1W rats transplanted with RT1-A-silenced cells showed to proliferate (Figure 6).

### 3.6. *In Vitro* Evaluation of NK Cell Activity

**Figure 7:**
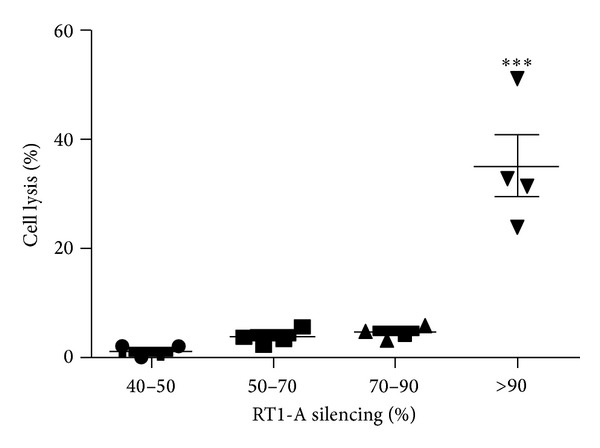
Natural killer (NK) cells cytotoxic assays. Freshly isolated NK cells of 4 rats were incubated with fibrosarcoma cells silenced for RT1-A expression at different levels at an effector : target (E : T) ratio 5 : 1 for 6 h. Cell lysis was detected by flow cytometry after staining with 7-amino-actinomycin D. Statistically significant differences are shown with asterisks (***P < 0.001).

Means of 1.05% ± 0.86 and 3.5% ± 1.33 of target cell lysis were achieved when 40–50% and 50–70% RT1-A silenced fibrosarcoma cells were used, respectively. A maximum of 5% of fibroblasts silenced by up to 90% for RT1-A expression were lysed by NK cells. The killing rates were significantly higher when fibrosarcoma cells expressing less than 10% of the normal RT1-A expression were used as target cells (34.94% ± 11.62, *P* < 0.001). Hence, for the allogeneic transplantation experiments we have used a fibrosarcoma cell line with a residual RT1-A expression of more than 10% as compared to the nonmodified cells or shNS-producing cells to prevent rejection due to NK cell activation (Figure 7).

## 4. Discussion

The variability of MHC genes is a major obstacle to allogeneic transplantation. Several studies have demonstrated a positive correlation between the number of donor-recipient MHC genes mismatches and the rate of rejection episodes after allogeneic transplantation. Previously, we demonstrated the feasibility of permanently silencing HLA class I and II expression in several human cell types. Other *in vitro *studies have shown that HLA-silenced cells are not only able to prevent the initiation of specific immune responses and are also protected from antibody- and cell-mediated cytotoxicity. Moreover, *in vitro *analyses have shown that functional properties of HLA-silenced cells were comparable to those of nonengineered cells [21–23]. In this study, we used an allogeneic transplantation model in rats to investigate whether MHC class I-silenced cells are able to engraft, proliferate, and survive *in vivo*. Regenerative medicine has opened up avenues for the development of novel therapeutic approaches to treat degenerative diseases and organ failure diseases, the incidence of which has risen significantly in the last few years. Human ESCs or iPS cells have gained much attention for their capacity to differentiate into cell types potentially involved in the pathogenesis of specific diseases [33, 34]. In fact, this strategy allows for the generation of disease-specific iPS cell lines for diseases such as Huntington's disease, congenital dyskeratosis, and fragile X syndrome [3, 35, 36]. However, MHC incompatibility remains a major hurdle for regenerative medicine as most of the developed products are of allogeneic origin.

This study showed that MHC class I-silenced cells are capable of escaping the immune response by preventing the activation of effector cells. Thus, molecular silencing of MHC class I expression may represent a new tool to facilitate the use of off-the-shelf products in regenerative medicine without the need for immunosuppression. Silencing of MHC expression may not only overcome the compatibility-related problems of MHC mismatch but also prevent the presentation of antigens derived from other polymorphic proteins (minor histocompatibility antigens). In our model, we have used a fibroblast cell line susceptible to T- and NK-cell activity. Here, transplantation of MHC class I-expressing cells using the haplotype-mismatched transplantation model resulted in complete cell loss in all animals within three weeks. Silencing of MHC class I expression was shown to be stable after cell transplantation during the monitored time period. Only one out of eight LEW.1W rats receiving allogeneic MHC class I- silenced cell transplants developed complete cell rejection six weeks after transplantation. In all other rats, the transplanted RT1-A silenced cells remained detectable until the end of the study (week 8). Although the cause of cell rejection in one of the animals is unclear, the fact that the cells were rejected at a later time may indicate for a chronic rejection mechanism that might have been triggered by MHC class II incompatibility between the donor and recipient cells. Presence of circulating donor-specific antibodies (DSA) has been recognized as a relevant cause of allograft rejection [37, 38]. IgG levels have been used to monitor the levels of DSA after transplantation [39]. We have evaluated rat IgG plasma levels after transplantation of MHC class I-silenced and nonsilenced cells in an allogeneic manner. We have observed significantly higher IgG plasma levels in animals transplanted with MHC expressing cells in comparison to the IgG levels detected in animals receiving MHC class I-silenced cells (Table S2). In this setting, IgG plasma levels clearly indicate for humoral rejection. However, the animal that lost the MHC class I-silenced cells at week 7 shows much lower IgG plasma levels than the rats that rejected the MHC class I-expressing cells at week 3. In comparison to the animals that did not rejected the MHC class I-silenced cells, only a slight increase in IgG levels was observed in the plasma of the only animal that lost the MHC class I-silenced cells. Hence, it is not possible to conclude that this animal lost the MHC class I-silenced cells only due to a humoral response. Nevertheless, also in this animal silencing MHC expression significantly extended graft survival as the transplanted MHC class I-silenced cells were rejected at a later time point.

 Regulation of NK cell activity requires a tight balance between activating and inhibitory signals, which is mediated by distinct classes of receptors on the cell surface. Activating signals are interrupted when inhibitory receptors on NK cells engage MHC class I molecules on target cells [40]. This receptor system is the basis for the “missing self-” activation mechanism enabling NK cells to detect virus-infected cells and cancer cells that express no MHC class I molecules, as proposed by Karre and colleagues [41]. We have determined the residual RT1-A expression level on the fibroblasts required to prevent NK cell lysis. The most efficient shRNA in silencing RT1-A expression without causing the activation of NK cells was selected for the transplantation experiments. Furthermore, we assessed NK cell infiltration in tissues generated from RT1-A silenced cells but observed no abnormal NK cell infiltration after either syngeneic or allogeneic transplantation. The absence of NK cell infiltration in MHC class I-silenced tissues shows that the residual MHC expression might be sufficient to prevent NK cell activation, as we previously demonstrated *in vitro* [22] and confirmed in this study. The fact that no T cells were detected and that proinflammatory cytokine levels were comparable to those detected after haplotype-matched transplantation also suggests a lower recruitment of effector cells to the graft.

Memory is a major characteristic of the adaptive immune system. Environmental antigens, pathogens, or alloantigens can trigger the activation of adaptive immune responses of naïve T cells upon T-cell receptor (TCR) interaction with specific complexes formed by antigens loaded into MHC molecules and presented by antigen-presenting cells (APCs). Antigen stimulation together with a costimulatory signal supplied by APCs triggers proliferation and subsequent differentiation of naïve T cells into effector T cells. Effector T cells secrete several cytokines and chemokines that recruit and activate auxiliary immune cells (e.g., B cells) for antibody production and macrophages for antigen clearance. It is not completely clear how many cell divisions cytotoxic T cells must undergo to acquire effector function. However, it is known that while naïve T cells differentiate into cytotoxic effector cells and they become capable of producing proinflammatory cytokines such as IFN-*γ* and TNF-*α* and cytotoxic molecules such as perforin and granzymes. In accordance with these observations, we detected a significant increase in serum cytokines (e.g., TNF-*α* and IFN-*γ*) in rats receiving allogeneic RT1-A-expressing cells in comparison to those treated with syngeneic or allogeneic RT1-A-silenced cells. Nonsilenced RT1-A haplotype-mismatched transplantation also resulted in a significant increase in IL-1*α* and IL-1*β* levels. The observed augmentation of IL-1 cytokine levels in animals during graft rejection is in accordance with the observations of others. Several studies have demonstrated that high levels of IL-1*α* and IL-1*β* are associated with T-cell infiltration [42, 43]. In this study, T-cell infiltration of the graft was positively associated with increased IL-1*α* and IL-1*β* levels. Most activated or effector T cells have a short lifetime, but a small percentage of activated cells persist and develop into long-lived memory T cells, which have the capacity to mediate potent recall responses when reactivated by antigens [44, 45]. In the present study, T-cell proliferation rates in rats transplanted with RT1-A-silenced cells were significantly lower after *in vitro *exposure to RT1-A-expressing cells than those in rats transplanted with RT1-A expressing cells. This observation indicates the presence of memory T cells in the animals receiving RT1-A-expressing grafts due to their faster capacity for recognition and proliferation after reexposure to the antigen *in vitro*. Conversely, the low proliferation rates exhibited by T cells derived from animals transplanted with RT1-A-silenced cells indicate that they had not been primed for the mismatched antigens.

In conclusion, the results of this study indicate that MHC class I silencing significantly prolongs graft survival following allogeneic transplantation. This effect is mainly due to the fact that MHC class I-silenced cells are not recognized by the recipient's immune system and therefore do not induce its activation. Silencing of MHC class I gene expression in grafted cells does not induce tolerance but “hides” the graft from the immune system. Silencing of MHC expression on transplanted cells, tissues, or organs has the potential to revolutionize the field of transplantation and to facilitate the re-expoadministration of many cell-based products developed for regenerative medicine purposes.

## Supplementary Material

The sequences of the shRNAs used in this study are provided in the Table S1. In addition, a description about the methods used for the determination of IgG plasma levels and the respective results are presented as supplementary information.

## Figures and Tables

**Table 1:** Cytokine secretion profile.

Analytes pg/mL	LEW (RT1-A cells)	LEW (RT1-A-silenced cells)	LEW.1W (RT1-A cells)	LEW.1W (RT1-A-silenced cells)
IL-1*α*	17.0 ± 5.9	15.5 ± 5.0	80.7 ± 20.3***	22.7 ± 10.3
IL-1*β*	23.2 ± 10.4	27.8 ± 9.2	130.9 ± 80.3***	32.1 ± 6.4
IL-6	8.0 ± 1.5	8.3 ± 2.1	45.9 ± 18.2***	10.3 ± 5.2
TNF-*α*	7.0 ± 3.2	7.2 ± 3.7	678.3 ± 345.4***	36.2 ± 29.8
IFN-*γ*	138.9 ± 58.7	126.2 ± 73.4	789.6 ± 111.2***	159.8 ± 123.6

Levels of different cytokines were measured in sera of LEW and LEW.1W rats transplanted with RT1-A-expressing fibroblasts (RT1-A cells) or RT1-A-silenced fibroblasts. Statistically significant differences are indicated by asterisks (***P < 0.001).
