# Supplementary material for: MHC Universal Cells Survive in an Allogeneic Environment after Incompatible Transplantation
Source: Biomed Res Int. 2013 Oct 9;2013:796046. doi: 10.1155/2013/796046 (PMC3856147; doi:10.1155/2013/796046)
Supplement: Supplementary file 1 — The sequences of the shRNAs used in this study are provided in the Table S1. In addition, a description about the methods used for the determination of IgG plasma levels and the respective results are presented as supplementary information. [file 796046.f1.pdf]

Supplementary Table 1

Name	Sequence
**shRNA_1**	5’ GACCGAGACATGTAATCAA -3’
**shRNA_2 (RN_shβ2m)**	5’ GGAAAGAAGATACCAAATA -3’

Supplementary Table 2

IgG (mg/ml)	Pre-Tx	Week 1	Week 2	Week 3	Week 4	Week 5	Week 6	Week 7	Week 8
**No rejection**<br><br>**MHC class I silencing cells (n=4)**	111.7±0.9	110.3±1.9	112.2±2.4	111.1±2.3	111.2±2.5	111.6±0.9	111.3±0.6	110.9±1.1	110.3±0.7
**Rejection**<br><br>**MHC class I silenced cells (n=1)**	111.3	110.7	112.8	112.4	112.6	112.8	112.6	112.5<br><br>(graft not detectable)	112.7
**Rejection**<br><br>**MHC class I expressing cells (n=3)**	111.2±0.7	112.9±0.2	114.1±0.9	116.9±2.9<br><br>(graft not detectable)	116.7±2.2	116.2±2.6	115.8±3.3	115.9±4.2	115.8±4.0
